# Superiority of long-term outcomes of EUS-guided gastroenterostomy over endoscopic stenting for the palliation of malignant gastric outlet obstruction: A prospective cohort study

**DOI:** 10.1097/eus.0000000000000196

**Published:** 2026-07-07

**Authors:** Yu-Ting Kuo, Sheng-Jie Chang, Hung-Yao Lin, Tung-Yen Lin, Weng Fai Wong, Ming-Lun Han, Chieh-Chang Chen, Shih-Hung Yang, Hsiu-Po Wang

**Affiliations:** 1 Division of Endoscopy, Department of Integrated Diagnostics & Therapeutics, National Taiwan University Hospital, National Taiwan University, Taipei, Taiwan, China; 2 Department of Internal Medicine, College of Medicine, National Taiwan University, Taipei, Taiwan, China; 3 Department of Internal Medicine, National Taiwan University Hospital Bei-Hu Branch, National Taiwan University College of Medicine, Taipei, Taiwan, China; 4 Division of Gastroenterology and Hepatology, Department of Internal Medicine, National Taiwan University Hospital, Taipei, Taiwan, China; 5 Division of Gastroenterology and Hepatology, Department of Internal Medicine, National Taiwan University BioMedical Park Hospital, Hsinchu, Taiwan, China; 6 Department of Internal Medicine, National Taiwan University Cancer Centre, National Taiwan University College of Medicine, Taipei, Taiwan, China; 7 Department of Oncology, National Taiwan University Hospital, Taipei, Taiwan, China; 8 Division of Gastroenterology, Center of Excellence for Innovation and Endoscopy in Gastrointestinal Oncology, Faculty of Medicine, Chulalongkorn University, Bangkok, Thailand.

**Keywords:** malignant gastric outlet obstruction, endoscopy, EUS-guided gastroenterostomy, metal stent

## Abstract

**Background and Objectives:**

Limited prospective data exist comparing long-term outcomes of endoscopic placement of a self-expandable metallic stent (ES) and EUS-guided gastroenterostomy (EUS-GE) for unresectable malignant gastric outlet obstruction (mGOO). This large-scale study aimed to prospectively compare these 2 procedures with long-term follow-up.

**Methods:**

This single-center, prospective study enrolled 152 consecutive patients with unresectable mGOO who underwent ES (*n* = 76) or EUS-GE (*n* = 76) between August 2021 and January 2024, with follow-up until death or administrative censoring on January 31, 2025. The primary outcome was the reintervention rate, while secondary outcomes included technical and clinical success, changes in the gastric outlet obstruction score (GOOS), adverse events (AEs), and survival. A Cox regression model identified factors associated with reintervention and survival.

**Results:**

Both groups had comparable technical and clinical success rates and AEs. After a median follow-up of 121 days (interquartile range, 55–285), the EUS-GE group had a significantly lower reintervention (adjusted hazard ratio, 0.22, *P* < 0.001), longer stent patency (median, 566 days *vs*. 195 days; *P* < 0.001), and greater GOOS improvement (median, 3 *vs*. 2; *P* < 0.001) compared with the ES group. Poor performance status, the presence of ascites, and lack of chemotherapy were independent mortality risk factors.

**Conclusion:**

Compared with ES, EUS-GE provides longer stent patency, fewer reinterventions, and better relief of GOO symptoms while maintaining a similar safety profile. In an expert setting, EUS-GE should be considered the optimal approach for managing unresectable mGOO, as it offers superior short- and long-term outcomes.

## INTRODUCTION

Malignant gastric outlet obstruction (mGOO), caused by luminal narrowing at the pylorus or duodenum, commonly affects patients with advanced pancreatobiliary or gastroduodenal cancers. It has been reported that approximately 15%–30% of patients with advanced pancreatic or periampullary malignancies develop mGOO.^[1,2]^ Common symptoms such as vomiting, early satiety, and weight loss can lead to malnutrition, delayed cancer treatment, and poor outcomes.^[3]^ Regardless of tumor origin, mGOO is associated with a poor prognosis.^[4]^

Traditionally, mGOO is managed with either endoscopic enteral stent placement (ES) or surgical gastroenterostomy (SGE). ES provides rapid symptom relief, lowers procedure risks, and shortens hospital stays; however, it carries a high reintervention rate (up to 33%) due to stent obstruction from tumor ingrowth or tissue overgrowth.^[5]^ In contrast, SGE offers more durable outcomes with fewer reinterventions but is associated with a higher risk of postoperative complications, such as gastroparesis.^[6]^ Current guidelines recommend ES for patients with less than 6 months’ life expectancy and SGE for those with longer prognosis and good performance status.^[7,8]^

EUS-guided gastroenterostomy (EUS-GE) is a novel, minimally invasive technique for bypassing mGOO using a lumen-apposing metal stent (LAMS).^[9]^ It combines the durability of SGE with the lower invasiveness of ES.^[10]^ Retrospective studies of over 500 EUS-GE cases report high technical (89%–99%) and clinical (82%–94%) success rates, with adverse events (AEs) in 16%–28% of cases.^[11]^ Additionally, a meta-analysis of 15 studies (1441 patients) found that EUS-GE had better clinical outcomes, fewer complications, and lower reintervention risks than both ES and SGE.^[12]^ However, most comparisons between ES and EUS-GE are retrospective and based on Western populations. To address this, we conducted the largest prospective cohort study with at least 1 year of follow-up to compare short- and long-term outcomes of ES and EUS-GE in mGOO patients.

## METHODS

This prospective cohort study was conducted at National Taiwan University Hospital in Taiwan, China. The study was approved by the Institutional Review Board of National Taiwan University Hospital (202108046RINC), and written informed consent was obtained from all enrolled patients. The trial was registered at ClinicalTrials.gov (NCT07230665). All authors had full access to the study data and reviewed and approved the final manuscript.

### Patients and procedures

Between August 2021 and January 2024, consecutive patients aged 18 years or older who underwent ES or EUS-GE for unresectable mGOO were enrolled. Exclusion criteria included prior enteral stent placement, prior gastric surgery, multilevel bowel obstruction, linitis plastica of the stomach, a life expectancy of less than 1-month, refractory coagulopathy or thrombocytopenia, pregnancy, and inability to provide informed consent. Life expectancy was determined by multidisciplinary team (MDT)—comprising oncologists, gastroenterologists, and surgeons—based on clinical status, tumor burden, Eastern Cooperative Oncology Group (ECOG) performance status, and the planned oncologic strategy. Following MDT consensus, patients with a limited life expectancy (<6 months), poor performance status, or a longer life expectancy (>6 months) who declined surgery were referred to endoscopic management. The final choice between ES and EUS-GE was determined through shared decision-making. Both procedures were discussed in detail, including procedural risks, expected benefits, and cost differences. Since EUS-GE is not fully reimbursed by the National Health Insurance system in Taiwan, the higher out-of-pocket expenditure for EUS-GE (approximately $7000 *vs*. $2000 for ES) was integrated into the patient’s final decision-making process. All procedures were performed by 2 experienced endoscopists (Y.T.K. and H.P.W.) in an endoscopy unit. Patients undergoing EUS-GE were performed under general anesthesia with endotracheal intubation, while those undergoing ES were under conscious sedation. For both EUS-GE and ES, the obstruction was accessed with either a duodenoscope or a therapeutic forward-viewing endoscope. A 0.025- or 0.035-inch guidewire was advanced into the jejunum beyond the stricture, after which contrast injection was performed to delineate its location and length before proceeding with the selected procedure.

### EUS-guided gastroenterostomy

For all EUS-GE procedures, patients received a single intravenous dose of prophylactic antibiotics. After delineating the obstruction, a 7Fr nasobiliary drain was advanced over a guidewire into the target jejunum under fluoroscopy. A linear echoendoscope was then advanced into the stomach to visualize the jejunum via EUS. The jejunal loop was adequately distended by continuously infusing a mixture of saline, contrast medium, and indigo carmine using a standard water pump. Once the target jejunum was confirmed, an antispasmodic (glucagon) was administered. Using the wireless EUS-GE simplified technique,^[13]^ the gastric and jejunal walls were directly punctured with an electrocautery-enhanced LAMS (Hot AXIOS, 20 mm diameter, 10 mm length; Boston Scientific, Marlborough, MA, USA). The LAMS was deployed under EUS and fluoroscopic guidance—first the distal flange into the jejunum, followed by intrachannel release of the proximal flange within the echoendoscope, and then its advancement outside the working channel.

### Endoscopic enteral stent placement

An uncovered, through-the-scope duodenal stent (BONASTENT; Standard Sci Tech, Seoul, Korea or WallFlex; Boston Scientific, Marlborough, MA, USA) with a diameter of 22 mm and a length of 6–16 cm, depending on the stricture length, was then deployed to adequately cover both ends of the obstruction under combined fluoroscopic and endoscopic guidance.

### Postprocedural care and long-term follow-up

After the procedure, all patients were followed according to a standardized protocol (Supplementary Table 1, https://links.lww.com/ENUS/A401) for up to 12 months or until death or administrative censoring (January 31, 2025). Oral refeeding with a clear liquid diet began 4–6 hours postprocedure and progressed to a liquid diet on postoperative day (POD) 1, a soft diet on POD 2, and a low-residue or normal diet from POD 3 if tolerated. At 1 month, patients were asked to report their weight and specify their oral intake. Follow-ups were conducted in the outpatient clinic every 3 months until death or January 31, 2025. Patients were advised to visit the emergency department or contact their physician if GOO symptoms recurred. For missed visits, physicians contacted patients or their families by telephone to inquire about emergency department visits, hospitalizations, current vital status, and, for deceased patients, the cause of death.

### Outcome assessments

The primary outcome was the reintervention rate, defined as the requirement for additional endoscopic procedures due to recurrent GOO symptoms after initial clinical recovery. Recurrent GOO was defined as recurrence of obstructive symptoms (such as nausea, vomiting, or intolerance of oral intake) accompanied by a decline in GOO scores (GOOSs). When clinically indicated, recurrence was confirmed by endoscopic or radiologic evaluation demonstrating stent dysfunction or obstruction before reintervention was performed. Reinterventions were classified as early if they occurred within 30 days of the index procedure. Notably, rescue interventions performed during the index procedure due to technical issues were excluded from the reintervention count. Secondary outcomes included technical success, clinical success, changes in GOOS, AEs, and survival. Technical success was defined as the successful placement of a stent across or bypassing the obstruction, as confirmed by endoscopy or fluoroscopy. Clinical success was defined as an improvement of ≥2 points in the GOOS compared with baseline, assessed daily postprocedure until the time of hospital discharge. The GOOS categorizes the ability to eat into 4 levels based on the highest tolerated intake: 0 (no oral intake), 1 (liquid diet), 2 (soft diet), and 3 (normal/low-residue diet).^[14]^ Postprocedural GOOS were recorded daily until discharge according to a standardized postoperative feeding protocol. The time to reach the maximum GOOS following the procedure was also documented for each patient. AEs were classified and graded according to the Adverse Events in GI Endoscopy classification,^[15]^ with a 30-day cutoff distinguishing early and late events.

Predictive factors for reintervention and survival were analyzed using body mass index classifications: underweight (<19 kg/m^2^), normal (19–24.9 kg/m^2^), overweight (25–29.9 kg/m^2^), and obese (≥30 kg/m^2^). Nutritional status was assessed by serum albumin: normal (≥3.5 g/dL), mild malnutrition (>3 to <3.5 g/dL), moderate malnutrition (>2.5 to <3 g/dL), and severe malnutrition (<2.5 g/dL). Preprocedural performance was evaluated using ECOG scales: 0–1 (no to mild limitation) and 2–4 (moderate to severe limitation).^[16]^ Receipt and timing of systemic chemotherapy after the procedure were systematically recorded and included as variables in the reintervention and survival analysis.

### Statistical analysis

Categorical variables were presented as frequencies with percentages and were compared using the Fisher’s exact test. Normally distributed continuous variables (age and albumin) were expressed as mean ± standard deviation and analyzed using the Student *t* test. Nonnormally distributed continuous variables (body mass index, interval from tumor diagnosis to GOO, procedure time, GOOS, hospital stays, follow-up duration, interval from procedure to reintervention and death) were reported as median with interquartile range (IQR) and compared using the Mann–Whitney *U* test. Cumulative stent patency and survival were evaluated using the Kaplan–Meier method and compared with the log-rank test. In Kaplan–Meier analyses of stent patency and time-to-reintervention, death without prior reintervention was treated as a censoring event. For reintervention analysis, time-to-first reintervention was used as the event variable. Cox proportional hazards regression model was performed to identify risk factors for reintervention and survival following EUS-GE or ES. Variables with a *P* value <0.2 in univariable analysis were included in the multivariable regression model. The proportional hazards assumption was assessed using Schoenfeld residuals. To account for the potential influence of postprocedural chemotherapy on reintervention risk, a sensitivity analysis using a 30-day landmark approach was performed, including only patients alive 30 days after the index procedure. Statistical analyses were performed using Stata version 15 (StataCorp, College Station, TX, USA). All tests were 2-tailed, with *P* <0.05 considered statistically significant. Assuming a reintervention rate of 14.5% after EUS-GE and a significance level of 0.05, a total of 76 patients per group would provide 99% power to detect a difference between EUS-GE and ES corresponding to a hazard ratio (HR) of 0.22 (nQuery Advisor 4.0, Statistical Solutions Ltd, Boston, MA, USA).

## RESULTS

**Figure 1.**
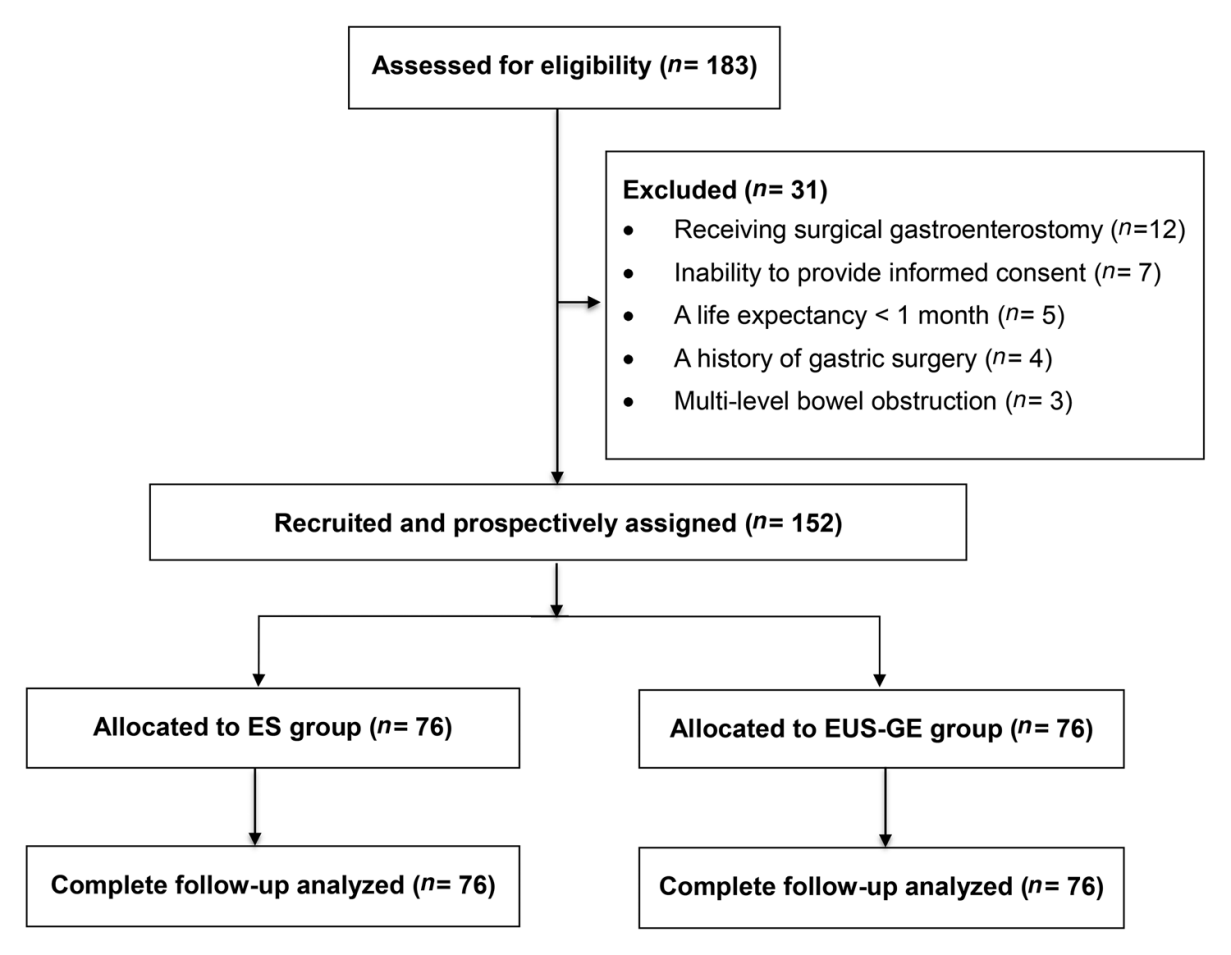
Flow diagram of patient enrollment. ES, endoscopic enteral stent placement; EUS-GE, EUS-guided gastroenterostomy.

Of the 183 consecutive patients assessed for endoscopic management of mGOO during the study period, 31 were excluded, and the remaining 152 patients were enrolled and underwent either ES (*n* = 76) or EUS-GE (*n* = 76) [Figure 1]. All allocated patients completed prospective follow-up and were analyzed. No significant differences were observed between the 2 groups in baseline characteristics [Table 1].

**Table 1:** Comparison of the baseline characteristics between the enteral stent group and EUS-guided gastroenterostomy group.

	ES (*N* = 76)	EUS-GE (*N* = 76)	*P* value
Age, years, mean ± SD	66.5 ± 12.6	67.1 ± 13.7	0.697
Male gender, *n* (%)	39 (51.3)	42 (55.3)	0.745
BMI, median (IQR)	20.6 (18.2–23.4)	20.1 (17.8–22.4)	0.346
Weight, *n* (%)			
Underweight	25 (32.9)	31 (40.8)	0.426
Normal weight	41 (53.9)	40 (52.6)	
Overweight	9 (11.8)	5 (6.6)	
Obese	1 (1.3)	0	
Albumin, g/dL, mean ± SD	3.4 ± 0.7	3.4 ± 0.6	0.906
Nutrition, *n* (%)			
Normal	33 (43.4)	33 (43.4)	0.263
Mildly malnourished	18 (23.7)	23 (30.3)	
Moderately malnourished	19 (25)	19 (25)	
Severely malnourished	6 (7.9)	1 (1.3)	
ECOG, *n* (%)			
None/mild	44 (57.9)	45 (59.2)	1
Moderate/severe	32 (42.1)	31 (40.8)	
Interval from tumor diagnosis to GOO, days, median (IQR)	236 (60–594)	185 (71–423)	0.332
GOO type, type 1/type2/type 3, *n* (%)	39 (51.3)/15 (19.7)/22 (28.9)	35 (46)/11 (14.5)/30 (39.5)	0.367
Presence of distal metastasis, *n* (%)	54 (71.1)	45 (59.2)	0.173
Presence of ascites, *n* (%)	36 (47.4)	41 (53.9)	0.517
Presence of peritoneal seeding, *n* (%)	23 (30.3)	24 (31.6)	1
Diagnosis, *n* (%)			
Pancreatic cancer	45 (59.2)	53 (69.7)	0.865
Cholangiocarcinoma	5 (6.6)	4 (5.3)	
GB adenocarcinoma	1 (1.3)	2 (2.6)	
Hepatocellular carcinoma	1 (1.3)	0	
Duodenal cancer	1 (1.3)	1 (1.3)	
Ampullary cancer	6 (7.9)	7 (9.2)	
Gastric cancer	8 (10.5)	4 (5.3)	
Genitourinary cancer	3 (3.9)	2 (2.6)	
Sarcoma	0	1 (1.3)	
Gynecologic cancer	2 (2.6)	1 (1.3)	
Breast cancer	1 (1.3)	0	
Colon cancer	3 (3.9)	1 (1.3)	
Cancer type, *n* (%)			
Pancreatic cancer	45 (59.2)	53 (69.7)	0.235
Nonpancreatic cancer	31 (40.8)	23 (30.3)	

BMI, body mass index; ECOG, Eastern Cooperative Oncology Group; ES, endoscopic enteral stent; EUS-GE, EUS-guided gastroenterostomy; GOO, gastric outlet obstruction score; IQR, interquartile range; SD, standard deviation.

### Short-term outcomes

As shown in Table 2, technical and clinical success rates were comparable between ES and EUS-GE (98.7% *vs*. 100% and 97.4% *vs*. 97.4%; *P* = 1 for both). However, the procedure time was significantly shorter with ES (median, 25 minutes [IQR, 18–33]) than EUS-GE (median, 35 minutes [IQR, 28–47]; *P* < 0.001). In the EUS-GE group, 1 patient experienced type 1 stent misdeployment (distal flange deployed in the peritoneum),^[17]^ which was successfully corrected in the same session.^[18]^ Two other patients had clinical failure due to occlusion of the contralateral jejunal wall by the distal flange of the LAMS, requiring additional duodenal stent placement through the LAMS. In the ES group, 1 patient had both technical and clinical failure due to unsuccessful guidewire passage, and another had clinical failure due to stent misdeployment, leaving the obstruction partially uncovered. GOOS improvement was significantly better in the EUS-GE group (median, 3 [IQR, 2–3]) compared with the ES group (median, 2 [IQR, 1–2]; *P* < 0.001), indicating superior symptom relief.

**Table 2:** Comparison of the outcomes between the ES group and EUS-GE group.

	ES (*N* = 76)		EUS-GE (*N* = 76)	*P* value
Short-term outcomes				
Procedure time, min, median (IQR)	25 (18–33)		36 (28–47)	<0.001
Technical success, *n* (%)	75 (98.7)		76 (100)	1
Clinical success, *n* (%)	74 (97.4)		74 (97.4)	1
Pretreatment GOOS, median (IQR)	0 (0–1)		0 (0–1)	0.977
Posttreatment GOOS, median (IQR)	2 (2–2)		3 (3–3)	<0.001
Change in GOOS, median (IQR)	2 (1–2)		3 (2–3)	<0.001
Hospital stays, days, median (IQR)	9 (5–18)		7 (4–13)	0.078
Treatment modality after procedure, *n* (%)				
Chemotherapy alone	33 (43.4)		49 (64.5)	0.055
Radiotherapy alone	4 (5.3)		1 (1.3)	
Combined both treatments	1 (1.3)		2 (2.6)	
No treatment	38 (50)		24 (31.6)	
Proportion of initiating chemotherapy within 30 days, *n* (%)	30 (88.2)		50 (98)	0.060
Interval from procedure to chemotherapy, days, median (IQR)	10 (7–14)		9 (6–14)	0.545
Early reintervention, *n* (%)	10		3 (3.9)	0.039
Tumor ingrowth	(13.2)		0	
Stent kinking	6 (7.9)		0	
Bleeding	3 (3.9)		1 (1.3)	
LAMS compression at the jejunal site	1 (1.3) 0		2 (2.6)	
Early adverse event, *n* (%)	2 (2.6)		2 (2.6)	1
Bleeding	1 (1.3)		1 (1.3)	
Stent misdeployment	1 (1.3)		1 (1.3)	
Long-term outcomes				
Follow-up duration, days, median (IQR)	100 (44–234)	130 (62–292)		0.266
Interval from procedure to reintervention, days, median (IQR)	195 (91–652)	566 (378-not reached)		<0.001*
Interval from procedure to death, days, median (IQR)	99 (44–290)	127 (61–285)		0.716*
Overall reintervention, *n* (%)	31 (40.8)	11 (14.5)		<0.001
Tumor ingrowth	18 (23.7)	1 (1.3)		
Tissue overgrowth	1 (1.3)	2 (2.6)		
Stent migration	0	1 (1.3)		
Stent fracture	4 (5.3)	0		
Stent kinking	5 (6.6)	0		
Bleeding	3 (3.9)	2 (2.6)		
Narrowing and lengthening of the LAMS	0	3 (3.9)		
LAMS compression at the jejunal site	0	2 (2.6)		
Overall adverse event, *n* (%)	4 (5.3)	3 (3.9)		0.699
Stent misdeployment	1 (1.3)	1 (1.3)		
Bleeding	3 (3.9)	2 (2.6)		
Perforation	0	0		
All-cause mortality, *n* (%)	69 (90.8)	70 (92.1)		1
Procedure-related mortality, *n* (%)	0	0		1

* Log-rank test.

ES, endoscopic enteral stent; EUS-GE, EUS-guided gastroenterostomy; GOOS, gastric outlet obstruction score; IQR, interquartile range; LAMS, lumen-apposing metal stent.

Reintervention within 30 days was significantly higher in the ES group compared with EUS-GE (13.2% *vs*. 3.9%, *P* = 0.039). Ten ES patients required additional duodenal stents due to tumor ingrowth (*n* = 6), stent kinking (*n* = 3), or bleeding from tumor ingrowth (*n* = 1). In the EUS-GE group, only 3 patients needed reintervention: 2 for distal jejunal angulation requiring additional duodenal stents, and 1 for suspected contralateral jejunal ulcer bleeding, requiring LAMS exchange. AEs within 30 days were comparable between groups.

### Long-term outcomes

**Figure 2.**
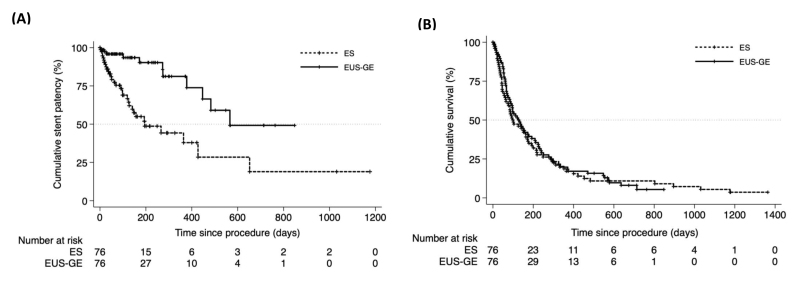
A, Kaplan–Meier curves for cumulative stent patency. Median stent patency was 566 days in the EUS-GE group and 195 days in the ES group, respectively (log-rank *P* < 0.001). B, Kaplan–Meier curves for cumulative patient survival. Median survival time was 127 days in the EUS-GE group and 99 days in the ES group, respectively (log-rank *P* = 0.716).

The long-term outcomes were summarized in Table 2. The median follow-up duration showed no significant difference between the ES group (100 days [IQR, 44–234]) and EUS-GE group (130 days [IQR, 62–292], *P* = 0.266). However, the EUS-GE group had a significantly lower overall reintervention rate (14.5% *vs*. 40.8%, *P* < 0.001) and a lower incidence of tumor ingrowth (1.3% *vs*. 23.7%, *P* < 0.001) compared with the ES group. Stent patency was significantly longer in the EUS-GE group (median, 566 days [IQR, 378–not reached] *vs*. 195 days [IQR, 91–652]; log-rank *P* < 0.001) [Figure 2A]. Clinical success rates in the EUS-GE group were also higher over 24 months (Supplementary Table 2, https://links.lww.com/ENUS/A402). Sixty-nine patients (90.8%) in the ES group and 70 (92.1%) in the EUS-GE group died from causes unrelated to the procedure (*P* = 1). No difference in overall survival was found between the 2 groups [log-rank *P* = 0.716; Figure 2B].

The Cox regression analysis results on factors associated with reintervention were summarized in Table 3. A total of 42 reintervention events were included in the Cox regression analysis evaluating predictors of reintervention. The analysis was performed using time-to-first reintervention. Multivariable analysis showed that EUS-GE was independently linked to a reduced risk of reintervention compared with ES (adjusted HR [aHR], 0.22; 95% confidence interval [CI], 0.11–0.44; *P* < 0.001). Poor performance status (ECOG score ≥ 2) (aHR, 2.35; 95% CI, 1.24–4.44; *P* = 0.009) and pancreatic cancer (aHR, 2.27; 95% CI, 1.12–4.58; *P* = 0.022) were associated with an increased reintervention risk. For survival predictors [Table 4], poor performance status (aHR, 1.74; 95% CI, 1.17–2.57; *P* = 0.006), the presence of ascites (aHR, 2.31; 95% CI, 1.59–3.39; *P* < 0.001), and the presence of distal metastasis (aHR, 1.49; 95% CI, 1.01–2.20; *P* = 0.042) were negative predictors, while receiving chemotherapy after the procedure was a significant positive predictor of survival (aHR, 0.52; 95% CI, 0.36–0.76; *P* = 0.001). In a sensitivity analysis using a 30-day landmark approach, restricted to patients alive beyond 30 days after the index procedure and accounting for postprocedural chemotherapy, EUS-GE remained significantly associated with a lower risk of reintervention (Supplementary Figures 1, https://links.lww.com/ENUS/A403, and 2, https://links.lww.com/ENUS/A404).

**Table 3:** Cox proportional hazards regression model for predicting the need for reintervention.

	Univariable analysis			Multivariable analysis		
	Hazard ratio	95% confidence interval	*P* value	Adjusted hazard ratio	95% confidence interval	*P* value
EUS-GE *vs*. ES	0.25	0.13–0.51	<0.001	0.22	0.11–0.44	<0.001
Sex, male/female	1.01	0.55–1.86	0.975	-	-	-
Age, per 1 year increase	1.01	0.99–1.04	0.296	-	-	-
Underweight *vs*. normal/overweight	0.76	0.39–1.46	0.407	-	-	-
Moderate/severe *vs*. none/mild nutrition	1.33	0.69–2.56	0.396	-	-	-
Moderate/severe *vs*. none/mild ECOG	1.75	0.95–3.24	0.074	2.35	1.24–4.44	0.009
Pancreatic *vs*. nonpancreatic cancer	1.74	0.87–3.46	0.117	2.27	1.12–4.58	0.022
Ascites, yes/no	0.83	0.44–1.56	0.577	-	-	-
Distal metastasis, yes/no	1.15	0.62–2.15	0.658	-	-	-
Peritoneal seeding, yes/no	0.72	0.35–1.47	0.365	-	-	-
Chemotherapy after procedure, yes/no	0.77	0.41–1.44	0.409	-	-	-

ECOG, Eastern Cooperative Oncology Group; ES, endoscopic enteral stent; EUS-GE, EUS-guided gastroenterostomy.

**Table 4:** Cox proportional hazards regression model for predicting survival.

	Univariable analysis			Multivariable analysis		
	Hazard ratio	95% confidence interval	*P* value	Adjusted hazard ratio	95% confidence interval	*P* value
EUS-GE *vs*. ES	0.94	0.67–1.32	0.733	1.16	0.80–1.69	0.421
Sex, male/female	0.85	0.61–1.18	0.329	-	-	-
Age, per 1 year increase	1.00	0.99–1.02	0.695	-	-	-
Underweight *vs*. normal/overweight	1.06	0.75–1.50	0.752	-	-	-
Moderate/severe *vs*. none/mild nutrition	1.33	0.93–1.92	0.120	1.21	0.81–1.80	0.359
Moderate/severe *vs*. none/mild ECOG	1.55	1.10–2.17	0.012	1.74	1.17–2.57	0.006
Pancreatic *vs*. nonpancreatic cancer	1.16	0.82–1.65	0.400	-	-	-
Ascites, yes/no	1.88	1.34–2.63	<0.001	2.31	1.59–3.39	<0.001
Distal metastasis, yes/no	1.61	1.12–2.30	0.010	1.49	1.01–2.20	0.042
Peritoneal seeding, yes/no	1.15	0.81–1.65	0.436	-	-	-
Chemotherapy after procedure, yes/no	0.58	0.42–0.82	0.002	0.52	0.36–0.76	0.001

ECOG, Eastern Cooperative Oncology Group; ES, endoscopic enteral stent; EUS-GE, EUS-guided gastroenterostomy.

## DISCUSSION

Prospective comparative data on the endoscopic management of unresectable mGOO remain limited, with only 2 published prospective studies to date.^[19,20]^ In this context, the present study represents the largest prospective cohort with a standardized follow-up protocol extending to 12 months. Although the median follow-up was inevitably constrained by the poor prognosis of this patient population, a subset of patients completed the full 12-month follow-up. This design enabled a robust comparison of efficacy, safety, and long-term outcomes between ES and EUS-GE. Our results demonstrate that EUS-GE provides superior stent patency and a lower reintervention rate compared with ES. Furthermore, EUS-GE achieved more effective relief of GOO symptoms while maintaining a safety profile comparable to ES. These findings offer high-quality prospective evidence to inform clinical decision-making in patients with incurable mGOO.

Current guidelines recommend SGE for patients with unresectable mGOO who have an expected survival of >6 months and adequate functional status.^[7,8]^ In our cohort, treatment strategies were determined through MDT. Although some patients were considered potential surgical candidates [*n* = 164; Figure 1], the majority declined SGE (*n* = 152; 92.7%), mainly because of concerns regarding postoperative recovery, procedural invasiveness, and potential delay in resumption of systemic oncologic therapy. Consequently, minimally invasive endoscopic approaches were often preferred in real-world practice. According to our standardized postprocedural protocol, oral intake was generally resumed within the first few days, with stepwise advancement from liquids to a soft diet. Effective relief of obstruction may therefore facilitate nutritional recovery and allow continuation of systemic therapy. In exploratory analyses, 94% patients [80/85; Table 2] were able to initiate systemic chemotherapy within 30 days after the index procedure, supporting the clinical rationale that successful endoscopic palliation may help maintain eligibility for oncologic treatment.

To date, the choice between uncovered self-expandable metal stents (U-SEMSs) and covered SEMSs for palliative endoscopic stent placement remains uncertain and should be based on regional stent availability.^[7,21]^ U-SEMSs are associated with a higher risk of stent occlusion, whereas covered SEMSs carry a greater risk of migration. However, there is no significant difference in overall stent patency or patient survival between the two.^[22,23]^ In our cohort, the median survival of mGOO patients after U-SEMS placement was 99 days, with nearly one-quarter requiring reintervention within 3 months [Table 2, Figure 2, and Supplementary Table 2, https://links.lww.com/ENUS/A402]. Notably, a randomized controlled trial (RCT) demonstrated that extending the follow-up duration from 8 to 16 weeks after U-SEMS placement for mGOO was associated with a 5-fold increase in the risk of stent obstruction, reaching approximately 40%,^[24]^ which aligns with our findings. Although ES is a less invasive procedure with faster recovery, high technical success, and a low AE rate, it may not be an ideal management option for mGOO due to its high stent dysfunction rate, leading to increased need for reintervention during long-term follow-up.

EUS-GE is a novel approach for bypassing mGOO using LAMS under EUS guidance, similar to the concept of SGE, in which the stent is placed away from the tumor. In contrast to ES, our study highlights that EUS-GE achieved comparable technical success (100% *vs*. 98.7%, *P* = 1) and similar rates of AEs (3.9% *vs*. 9.2%, *P* = 0.327) but significantly reduced the need for reintervention by nearly two-thirds (14.5% *vs*. 40.8%, *P* < 0.001) while also improving eating function (change in GOOS; median, 3 [IQR, 2–3]) *vs*. 2 [IQR, 1–2]; *P* < 0.001) after long-term follow-up. Additionally, in the multivariate Cox regression analysis, EUS-GE was identified as an independent factor associated with a nearly 80% reduction in the risk of reintervention compared with ES. Consistently, a recent RCT demonstrated that EUS-GE with the EPASS technique provided longer stent patency without obstruction and significantly improved the 1-month GOOS compared with ES, though follow-up was limited to only 6 months.^[19]^ Furthermore, 2 large multinational retrospective studies showed that EUS-GE is associated with better patient-reported eating habits and a lower risk of reintervention while maintaining similar safety.^[25,26]^ Moreover, a recent network meta-analysis of 4 RCTs and 36 non-RCTs comparing 4 palliative treatments for mGOO found that EUS-GE was more advantageous than both SGE and ES in terms of combined safety and efficacy.^[27]^ Taken together, these findings support EUS-GE as the optimal approach for achieving both short- and long-term outcomes in mGOO management. When interpreting our findings in the context of previous studies, it is important to note several methodological differences. For example, the DRA-GOO RCT evaluated EUS-GE performed using the EPASS technique, whereas all procedures in our cohort were performed using the wireless EUS-GE simplified technique. In addition, clinical success and postprocedural GOOS were assessed daily until discharge, and the time to achieve the maximum GOOS was recorded for each patient. In contrast, the timing of outcome assessment varied across prior studies. These differences should be considered when making comparisons between studies.

Because the mean life expectancy of patients with mGOO is typically short, GOO is considered a poor prognostic indicator of survival in malignancies, regardless of the type.^[4,28–30]^ In our study, the overall median survival after stent placement was 113 days, which aligns with the reported poststent placement survival less than 3 months in North America and Europe.^[4,28–30]^ However, we still explored potential factors influencing the survival prognosis of patients with unresectable mGOO. While neither ES nor EUS-GE, after relieving obstruction, was identified as an independent prognostic factor in the multivariate analysis, we found that poor performance status, the presence of ascites, distal metastasis, and the absence of postprocedural chemotherapy were independently associated with worse prognosis. These results were consistent with numerous studies reporting on various predictors of survival postmanagement for mGOO.^[4,31,32]^ Possible explanations include aggressive tumor biology or a large tumor burden, which may lead to poor prognosis despite stent patency.

This study has several limitations. First, the therapeutic modality for mGOO relief was not randomized. Because the choice of treatment was guided by shared decision-making and influenced by procedural costs, inherent selection bias and socioeconomic confounding cannot be entirely excluded. Nevertheless, all patients underwent a standardized MDT discussion and received counseling from the same group of endoscopists, thereby reflecting real-world clinical practice. Second, although a standardized follow-up protocol and Cox proportional hazards regression model were applied to reduce potential bias, multivariable adjustment in an observational study cannot completely eliminate residual confounding. In addition, because patients with unresectable mGOO generally have limited survival, the high competing risk of mortality may impact the interpretation of long-term outcomes, such as stent patency and time to reintervention. Third, although there was no significant difference in postprocedural oncologic treatment between the 2 groups, a higher proportion of patients receiving chemotherapy after EUS-GE was observed. As postprocedural chemotherapy may influence both survival and the likelihood of reintervention, additional sensitivity analyses adjusting for postprocedural chemotherapy were performed. The results were consistent with the primary analysis, with EUS-GE remaining significantly associated with a lower risk of reintervention compared with ES, supporting the robustness of this finding. Finally, this was a single-center study, and all procedures were performed by 2 experienced endoscopists with extensive expertise in therapeutic EUS. Therefore, the generalizability of these findings to less experienced endoscopists remains uncertain. Despite these limitations, a key strength of this study is that it represents the largest prospective cohort evaluating the efficacy, safety, and long-term outcomes of ES and EUS-GE in patients with mGOO.

In conclusion, EUS-GE in an expert setting can provide not only longer stent patency and requires fewer reinterventions but also offers better relief of GOO symptoms while maintaining a safety profile similar to ES. EUS-GE should be considered as the optimal approach for achieving both short- and long-term outcomes in unresectable mGOO management. However, given the observational nature of this study and the potential for residual confounding, our findings should be interpreted with caution. Further multicenter prospective studies are warranted to confirm these results.

## Supplementary Information

Supplemental digital content is available for this article. Direct URL citations are provided in the HTML and PDF versions of this article on the journal’s Web site (www.eusjournal.com).

## Ethical Statements

This study was approved by the Ethics Committee of National Taiwan University Hospital (202108046RINC) and registered at ClinicalTrials.gov (NCT07230665). The URL for the trial’s registry: https://clinicaltrials.gov/study/NCT07230665. Informed consents were obtained from all participants.

## Source of Funding

None declared.

## Conflicts of Interest

The authors declare no conflicts of interest.

## Author Contributions

Y.-T. Kuo and H.-P. Wang designed the study. Y.-T. Kuo prepared the statistical analysis. Y.-T. Kuo, S.-J. Chang, H.-Y. Lin, T.-Y. Lin, W. F. Wong, M.-L. Han, C.-C. Chen, S.-H. Yang, and H.-P. Wang recruited patients to the study. Y.-T. Kuo drafted the article, which was critically revised by H.-P. Wang. All authors commented on drafts and approved the final version. All authors had full access to the data and participated in the decision to submit for publication.

## Data Availability Statement

The data that support the findings of this study are available from the corresponding author upon reasonable request.
